# Genome Sequence of Staphylococcus pettenkoferi Strain SMA0010-04 (UGA20), a Clinical Isolate from Siaya County Referral Hospital in Siaya, Kenya

**DOI:** 10.1128/MRA.01626-18

**Published:** 2019-04-25

**Authors:** Vincent Otieno, Gary Xie, Qiuying Cheng, Hajnalka Daligault, Karen Davenport, Cheryl Gleasner, Lindsey Jacobs, Jessica Kubicek-Sutherland, Tessa LeCuyer, Evans Raballah, Norman Doggett, Harshini Mukundan, Benjamin McMahon, Douglas J. Perkins

**Affiliations:** a University of New Mexico Laboratories of Parasitic and Viral Diseases, Kisumu, Kenya; b Biosecurity and Public Health, Bioscience Division, Los Alamos National Laboratory, Los Alamos, New Mexico, USA; c Department of Internal Medicine, Center for Global Health, University of New Mexico Health Sciences Center, Albuquerque, New Mexico, USA; d Theoretical Biology and Biophysics, Theoretical Division, Los Alamos National Laboratory, Los Alamos, New Mexico, USA; e Physical Chemistry and Applied Spectroscopy, Chemistry Division, Los Alamos National Laboratory, Los Alamos, New Mexico, USA; f Department of Medical Laboratory Sciences, School of Public Health, Biomedical Sciences and Technology, Masinde Muliro University of Science and Technology, Kakamega, Kenya; Loyola University Chicago

## Abstract

Here, we report the sequence of a Staphylococcus pettenkoferi clinical isolate, strain SMA0010-04 (UGA20), which contains the PC1 beta-lactamase (*blaZ*) gene.

## ANNOUNCEMENT

Staphylococcus pettenkoferi, a Gram-positive human skin commensal bacterium, is a coagulase-negative staphylococcal species first described and associated with clinical specimens in 2002 (1). Since then, several additional cases of infection caused by this species have been reported in various countries around the world (2–5). Similar to other coagulase-negative staphylococci, S. pettenkoferi only rarely causes disease but may occasionally cause infection in patients whose immune system is compromised. The draft genome sequence of S. pettenkoferi strain SMA0010-04 (UGA20) reported here was isolated from the venous blood of a febrile female pediatric patient (20.0 months) at the Siaya County Referral Hospital in western Kenya in 2004. A laboratory test revealed that the patient was HIV negative with Plasmodium falciparum malaria and thrombocytopenia.

Upon admission, prior to any treatment interventions, blood was collected into a pediatric Isolator 1.5 microbial tube (Wampole Laboratories, Cranbury, NJ, USA) and cultivated at 35°C for 18 to 24 hours in 5% CO_2_ on 5% sheep blood agar. Bacterial DNA was extracted from a pure culture using the UltraClean microbial DNA isolation kit (Qiagen, Germantown, MD, USA) according to the manufacturer’s instructions with minimal modifications. The library was prepared from 100 ng of bacterial DNA using a NEBNext Ultra DNA library prep kit for Illumina (New England Biolabs, Ipswich, MA, USA). S. pettenkoferi SMA0010-04 (UGA20) was draft sequenced to 217-fold coverage using a MiSeq v2 500-cycle sequencing kit (Illumina, San Diego, CA, USA), resulting in 4,472,092 paired-end 251-bp reads. We were able to use BWA version 0.7.2 (6) to map SMA0010-04 (UGA20) reads covering 92.45% of the Staphylococcus pettenkoferi strain FDAARGOS_288 chromosome (GenBank accession number CP022096). Data quality was assessed, and the data files were filtered and trimmed with FaQCs version 1.3 (7) and then assembled with Velvet version 1.2.08 (8, 9) and IDBA version 1.1.0 (10). The consensus sequences were computationally shredded and reassembled with Phrap version SPS-4.24 (11, 12) to allow some manual editing with Consed (13), resulting in a final number of 158 (97.14% of the reads) contigs of >200 bp with an *N*_50_ value of 120,153 bp for SMA0010-04 (UGA20). The draft genome of SMA0010-04 (UGA20) consists of 2,519,169 bp, with an average G+C content of 38.7%. Annotations were completed at Los Alamos National Laboratory (LANL) using an automated system using the Ergatis workflow manager version 2.0 (14) and in-house scripts.

There are 2,491 predicted protein-coding genes, 64 tRNA genes, and 9 rRNA genes within the genome of SMA0010-04 (UGA20). Of these genes, 38% of the protein-coding genes were annotated in a SEED subsystem (15), while 62% were not annotated in a SEED subsystem. A total of 776 genes were annotated as hypothetical proteins. Of all the predicted genes, 2,249 are in common between SMA0010-04 (UGA20) and the Staphylococcus pettenkoferi strain FDAARGOS_288 genome, with 242 and 90 genes being unique to SMA0010-04 (UGA20) and FDAARGOS_288, respectively. Among them, the PC1 beta-lactamase (*blaZ*) gene unique in SMA0010-04 (UGA20) is associated with beta-lactam resistance, while 2 copies of the rRNA adenine *N*-6-methyltransferase (*ermA*) gene, unique in the FDAARGOS_288 strain, are associated with bacterial resistance to lincosamide, macrolide, and streptogramin (16).

### Data availability.

The GenBank accession number for Staphylococcus pettenkoferi SMA0010-04 (UGA20) is NWTY00000000, the BioProject accession number is PRJNA407945, and the BioSample accession number is SAMN07666372.
